# The use of locking plates in complex midfoot fractures

**DOI:** 10.1308/003588412X13373405386736

**Published:** 2012-05

**Authors:** E Bayley, N Duncan, A Taylor

**Affiliations:** Nottingham University Hospitals NHS Trust,UK

**Keywords:** Foot injuries, Fractures, comminuted, Tarsal bones, Fracture fixation

## Abstract

**INTRODUCTION:**

Complex fracture dislocations of the midfoot are uncommon. Improved outcomes have been demonstrated where it has been possible to restore and maintain the length and alignment of the medial column as well as the congruity of the articular surfaces. We present our experience with the use of angle-stable locking plates in the stabilisation of complex midfoot fracture dislocations.

**METHODS:**

Twelve patients were identified on a prospective trauma database between 2003 and 2009. All fractures involved the medial column with four associated fracture subluxations of the lateral column also. Patients underwent open reduction internal fixation (ORIF) with restoration of the medial column axis, reduction of the articular surface congruity and stabilisation with angle-stable locking plates.

**RESULTS:**

There were no post-operative infections or neurological injuries. Ten of the twelve patients required metalwork removal. There were no implant failures prior to removal of the metalwork. At a mean follow-up of 12.4 months (range: 4–32 months), 11 patients had minimal symptoms of swelling, discomfort or stiffness in the midfoot. This did not restrict their daily activities. One patient developed post-traumatic arthritis and collapse of the medial longitudinal arch. Two patients declined removal of the metalwork.

**CONCLUSIONS:**

Angle-stable locking plates provide satisfactory stabilisation following ORIF of complex midfoot fracture dislocations. Most patients will require removal of the metalwork. Following removal of metalwork, the majority of patients will maintain the length, alignment and stability of the midfoot.

Significant fractures and dislocations of the midfoot are unusual injuries. They constitute 0.1–0.9% of all fractures.^1–3^ Management can be difficult because of injury to a relatively poor soft tissue envelope combined with multifragmentary fractures of the bone segments that preclude anatomical reduction and intrafragmentary compression. In addition, injuries to the restraining ligaments and soft tissues may be surgically unreconstructable and yet render the midfoot unstable despite adequate reduction and fixation of the bony skeleton.

The aim of surgery is to restore length and alignment of the midfoot, and therefore maintain the relationship between the hind, mid and forefoot.^4–6^ Where possible, restoration of articular surfaces and joint congruity is desired. The reduction is stabilised using some form of fixation. Conventional surgery includes the use of K-wires, screws, plates and internal fixation.

Each of the fixation techniques has limitations. K-wires are placed percutaneously but they provide limited stabilisation,^7^ require transfixion of the articular surfaces and may be prone to infection. Similarly, external fixation devices provide limited stability, may be associated with pin site infections^8^ and poor patient compliance.

In 2003 temporary bridge plating of the medial column was described.^9^ The authors recommended this technique for comminuted fractures of the navicular. The aim was to restore length and alignment of the medial column by indirect reduction and a bridging technique. The internal fixation was removed once the fracture had consolidated to allow restoration of normal function of the transverse tarsal joint.

The limitations of conventional plate and screw fixation include the limited space available for the placement of screws in the intact bone segment to allow stable fixation. This may lead to loosening and pull-out of the screws, with subsequent loss of stability and fracture fixation.^10^ Damage to the underlying periosteal blood supply may also be a feature of standard screws and plate constructs.^10,11^

The potential advantage of angle-stable locking plate devices has been recognised elsewhere.^12,13^ These plates do not require intimate contact with the underlying bony skeleton. This minimises the potential for malalignment from a poorly contoured plate. The locked screw construct confers additional stability. This may be a particular advantage in areas where there is limited space for the application of screws and bone fragments such as the head of the talus when bridging the talonavicular joint. In addition, the construct may protect the underlying soft tissue envelope because intimate contact between the plate and bone is not required. We have applied the principals of locking plate fixation in the management of complex fracture dislocations of the midfoot. We present our experience of this in 12 patients.

## Methods

A review of a prospectively collected audit database over a six-year period (2003–2009) was performed. During this period, 12 patients (5 male, 7 female) were identified as having complex fracture dislocations of the midfoot. All presented to the emergency department on the day of injury. The mean age was 41.9 years (range: 19–21 years).

Eleven of the patients sustained high energy injuries while the other sustained the injury following a fall from a standing height. Of the eleven high energy injury cases, four had sustained falls from height, one was a pedal cycle injury and six were vehicle occupants in road traffic collisions.

All 12 patients suffered complex fractures involving the medial column of the midfoot (navicular and cuneiform, with or without involvement of the tarsometatarsal joints, with disruption of the talonavicular, navicular-cuneiform or intercuneiform joints.) Four had also sustained injuries to the lateral column of the ipsilateral foot (calcaneocuboid fractures and fracture subluxations). The fracture patterns, method of fixation and outcomes are listed in Table 1. Navicular fractures were classified according to Sangeorzan *et al*.^5^

**Table 1:** Summary of fractures sustained, treatment and outcome

Fracture pattern	Number of patients	Age (years)	Treatment	Removal of metalwork?	follow-up duration (months)	Outcome
Calcaneus fracture, comminuted cuboid fracture +/- fractures of navicular, cuneiforms or MT bases	2	55, 61	Lateral column locking plate +/- lag screw to calcaneus, navicular or cuneiforms	Yes (1)	12, 24	Restricted inversion/eversion, minimal pain; CC ankylosis in the patient with retained metalwork
Type 3 navicular fracture, TN sub-luxation +/- CC subluxation	8	19-81	Medial column locking plate + external fixation lateral column in 1 patient	Yes (all)	4-32	Slight stiffness, slight discomfort or mild swelling; 1 patient: post-traumatic arthritis, medial arch orthotics
Lisfranc injury, base of medial cuneiform fracture, navicular-cuneiform subluxation	1	44	Locking plate medial cuneiform - 1st MT, lag screw to cuneiform fracture, positional screw to Lisfranc joint	Yes	13	Pain free, slight stiffness
Calcaneus fracture, ST dislocation, TN and CC subluxation, type 3 navicular fracture, large soft tissue defect	1	21	Medial column locking plate, primary fusion of ST joint, latissimus dorsi flap	No	8	No pain from midfoot, discomfort and swelling from soft tissue reconstruction

TN = talonavicular; CC = calcaneocuboid; ST = subtalar; MT = metatarsal

All patients were admitted. Closed manipulation of fracture dislocations was undertaken to realign the foot and relieve the tension on the overlying soft tissues. Patients were placed in a temporary backslab incorporating an A-V Impulse System® compression device (Orthofix, Bussolengo, Italy). Surgery was undertaken after soft tissue swelling had subsided, judged by the overall appearance of the foot and the reappearance of skin wrinkles.

Surgery was performed using a thigh tourniquet. Fractures were approached via a dorsal or medial longitudinal incision. Reconstruction of the navicular was performed where possible to restore joint congruity and provide interfragmentary compression where appropriate. The intercuneiform and tarsometatarsal joints were reduced and stabilised at the same time. A bridging plate was applied across all injured segments, spanning from the talus to the cuneiform or first metatarsal where necessary. A 3.5mm reconstruction locking compression plate (Synthes, Welwyn Garden City, UK) was used. After appropriate contour, standard screws were used to apply the plate to the proximal and distal segments. Locking screws were then used to complete the stabilisation.

The patients were placed in a below knee cast and were kept touch weight bearing for the first six weeks. At this stage, the patients were mobilised out of cast, non-weight bearing, for a further six weeks. The bridging plate was removed at a median time of three months post-operatively (range: 2–6 months).

**Figure 1:**
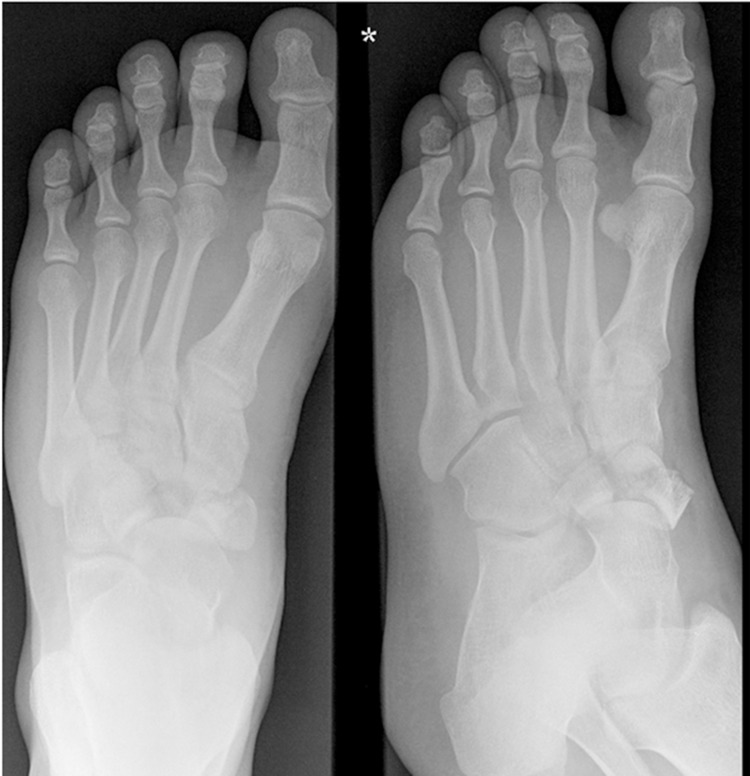
Pre-operative radiograph of a comminuted navicular fracture

**Figure 2:**
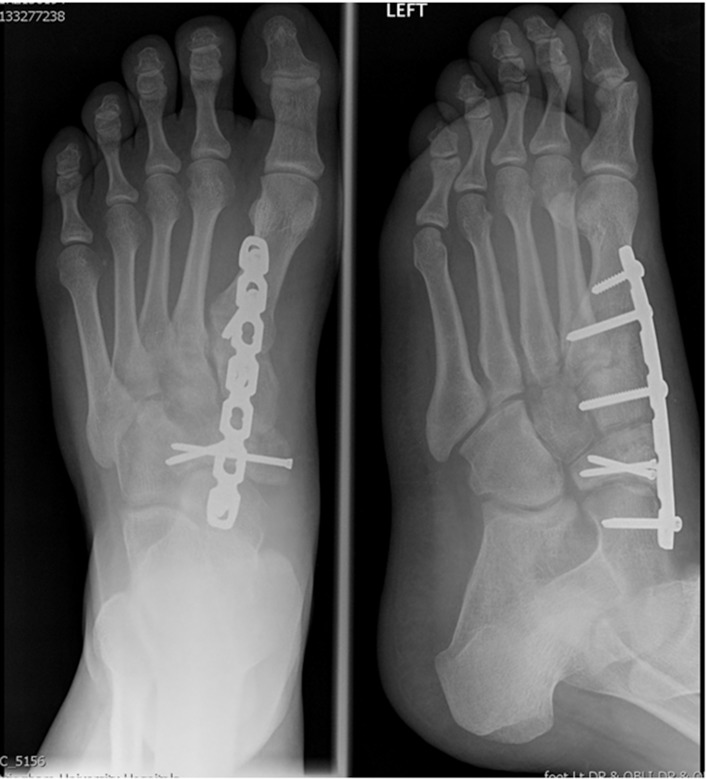
Radiograph following open reduction internal fixation with medial column locking plate

**Figure 3:**
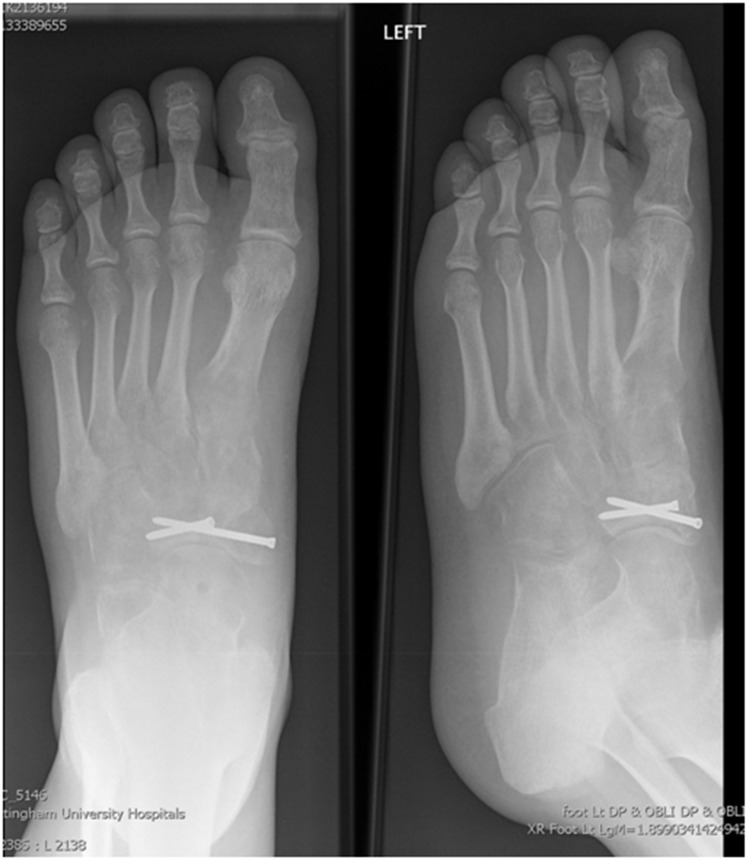
Weight bearing radiograph following removal of the bridge plate

An example of the management of a comminuted navicular fracture is demonstrated in Figures 1–3.

## Results

Patients were followed up in the outpatient fracture clinic for a mean duration of 12.4 months (range: 4–32 months) from the date of primary fracture fixation. There were no deep infections or acute neurological injuries. Ten of the twelve patients had their plate removed through the original incision. Two of the patients declined further surgery.

One patient developed post-traumatic collapse of the medial column and arthritis affecting the joints. This patient had had the metalwork removed at three months following fracture fixation. The changes were noted at a routine follow-up appointment ten months after the original surgery and did not occur prior to removal of the plate. This was managed non-operatively with a custom made functional foot orthosis.

The other eleven patients complained of either a minor degree of discomfort or stiffness at the midfoot. With regard to the two patients with retained metalwork, neither complained of prominence or pain specific to this.

Apart from one patient, none of the patients complained of restriction to their normal daily activities. (The one particular patient was describing symptoms from the soft tissue reconstruction but not specifically from the midfoot or retained metalwork).

## Discussion

Complex fracture dislocations to the midfoot are difficult to treat. They are often associated with a poor outcome.^6,7^ Treatment is aimed at anatomical reduction, fixation of displaced fractures, realignment of joints and articular surfaces, and achievement of stable fixation. Improved outcomes are associated with restoration of the articular surfaces as well as maintenance of the length of the medial and lateral columns.^4–6^

In an effort to improve stabilisation of the medial column after reduction, techniques describing bridge plating have been described. Schildhauer *et al* recommended spanning the medial column from the talus to the first metatarsal.^9^ However, conventional plates and screw techniques can be associated with a secondary loss of reduction following implant loosening.^10^

Angle-stable locking plate technology has gained widespread favour in the fixation of many fractures. These act as a fixed-angle device, and have been demonstrated to increase the bone implant construct’s stability in axial and torsional loading when compared with conventional plates and screws.^11,14–16^ In the foot and ankle, simulated calcaneal fracture fixation using locking plates has been shown to provide greater stability than conventional plate and screw techniques.^17^ The pull-out strength of individual screws is also increased compared with conventional implants.^10,11,14^ Where space is limited for fixation in the foot, this improved pull-out strength of the locking plate screws compared with conventional screws is likely to enhance stabilisation by reducing the risk of screw toggle, loosening and backing out of the screw.

Locking plates also have a potential advantage in fracture healing by reducing the footprint of the plate on the bone. This has been demonstrated to reduce the adverse affect on the periosteal soft tissues and blood supply.^10–12,14^ Where blood supply is tenuous (eg in the navicular), there is a further theoretical advantage to using a locking plate in that it does not require intimate contact with the underlying bone segments.

The potential advantages of locking plate technology and techniques prompted their use in this group of patients. In this series, there were no episodes of loss of reduction before the removal of metalwork despite the mobilisation of patients prior to fracture and soft tissue healing and consolidation.

A significant disadvantage of bridging plate techniques is that by spanning the joints, particularly at the transverse tarsal joint, movement through the midfoot is restricted to a significant extent.^6,18^ Consequently, it is recommended that the removal of metalwork is undertaken once the fracture and soft tissue healing is evident clinically and radiologically. In our series, there were no complications associated with the removal of metalwork such as infection or neurological injury. We therefore conclude that the potential advantage of locking plates over non-bridging techniques outweigh the potential risks of further surgery for metalwork removal.

We have reported a small number of patients in the study, comparable with other reports on these injuries.^8,9,19,20^ Studies of larger numbers of patients have been reported, gathering patients over longer time periods.^4,7^ This reflects the rare nature of these injuries. We believe that our report demonstrates the advantages locking plates can offer when managing complex fractures and dislocations of the midfoot, where reduction and fixation of the fracture alone it is not possible or insufficient to stabilise the foot.

## Conclusions

Following open reduction and internal fixation of complex fracture dislocations of the midfoot, locking plates provide adequate stabilisation with no evidence of loss of reduction during the healing process. The majority of patients will require removal of the metalwork. Following removal of the metalwork, satisfactory maintenance of length, alignment and stability of the midfoot is maintained, with satisfactory restoration of midfoot function following metalwork removal.
